# P29 Treatment outcomes with rezafungin and caspofungin in people aged 65 years and above with candidaemia and/or invasive candidiasis: integrated analysis of pooled Phase 2 and Phase 3 data

**DOI:** 10.1093/jacamr/dlad143.033

**Published:** 2024-01-03

**Authors:** Oliver A Cornely, George R Thompson, Alex Soriano, Bart-Jan Kullberg, Marin Kollef, Jose Vazquez, Patrick M Honore, Matteo Bassetti, John Pullman, Cecilia Dignani, Anita F Das, Taylor Sandison, Peter G Pappas

**Affiliations:** University of Cologne, Faculty of Medicine and University Hospital Cologne, Department of Internal Medicine, Excellence Center for Medical Mycology (ECMM), Cologne, Germany; University of Cologne, Faculty of Medicine and University Hospital Cologne, Chair Translational Research, Cologne Excellence Cluster on Cellular Stress Responses in Aging-Associated Diseases (CECAD), Cologne, Germany; University of Cologne, Faculty of Medicine and University Hospital Cologne, Clinical Trials Centre Cologne (ZKS Köln), Cologne, Germany; German Centre for Infection Research (DZIF), Partner Site Bonn-Cologne, Cologne, Germany; University of California Davis Medical Center, Sacramento, CA, USA; Hospital Clínic de Barcelona, IDIBAPS, University of Barcelona, Barcelona, Spain; Radboud University Medical Center, Nijmegen, The Netherlands; Washington University, St Louis, MO, USA; Augusta University, Augusta, GA, USA; Brugman University Hospital, Brussels, Belgium; University of Genoa, Genoa, Italy; Mercury Street Medical, Butte, MT, USA; PSI-CRO, Durham, NC, USA; Cidara Therapeutics Inc., San Diego, CA, USA; Cidara Therapeutics Inc., San Diego, CA, USA; University of Alabama at Birmingham, Birmingham, AL, USA

## Abstract

**Background:**

Factors including frailty and multimorbidity can affect candidaemia and/or invasive candidiasis (C/IC) treatment in older people.^1^ The current analysis explored data from C/IC patients aged ≥65 years who were treated with rezafungin or caspofungin in the STRIVE (Phase 2: NCT02734862) and ReSTORE (Phase 3: NCT03667690) clinical trials.^2,3^

**Methods:**

STRIVE and ReSTORE were double-blind, randomized studies. Adults with C/IC, diagnosed by systemic signs and mycological confirmation, received rezafungin once-weekly (Week 1: 400 mg; Weeks 2–4: 200 mg) or once-daily caspofungin (Day 1: 70 mg; Days 2–28: 50 mg) by IV injection for ≥14 days (≤4 weeks). Post hoc analysis examined pooled STRIVE/ReSTORE data for subjects aged ≥65 years. Safety outcomes included treatment emergent adverse events (TEAEs) and serious adverse events (SAEs) in subjects who received ≥1 dose of study drug (safety population). Day 30 all-cause mortality (ACM) and mycological response at Days 5 and 14 were examined for the modified intention-to-treat (mITT) population (subjects with mycological C/IC diagnosis within 96 h of randomization who received ≥1 study drug dose).

**Results:**

The safety population included 132 subjects (rezafungin arm: 64; caspofungin arm: 68). The mITT population included 120 subjects (rezafungin arm: 57; caspofungin arm: 63). The most common TEAEs with rezafungin were hypokalaemia, diarrhoea, vomiting and anaemia (Table 1). Eight subjects reported rezafungin-related TEAEs and seven had caspofungin-related TEAEs. SAEs comprised one case each of first degree atrioventricular block (rezafungin arm) and acute liver injury (caspofungin arm). Day 30 ACM rate was 14.0% (rezafungin arm) and 31.7% (caspofungin arm). The between-group difference (95% CI) was -17.6 (−32.5, −2.8). Day 5 mycological response was 78.9% (rezafungin arm) and 58.7% (caspofungin arm; difference [95% CI]: 19.3 [3.3, 35.2]; Figure 1).

**Conclusions:**

Integrated analysis of pooled STRIVE/ReSTORE study data revealed similar incidence of drug-related TEAEs and SAEs in patients aged ≥65 years treated with rezafungin or caspofungin. Further analyses are required to understand underlying factors influencing between-group differences regarding treatment outcomes.

**Table 1.** Safety data for candidaemia/invasive candidiasis patients aged ≥65 years treated with rezafungin (400 mg/200 mg) or caspofungin (70 mg/50 mg) (safety population)

	Rezafungin (400/200 mg) (*N*=64), *n* (%)	Caspofungin (70/50 mg) (*N*=68), *n* (%)
Subjects with at least 1 TEAE	59 (92.2)	62 (91.2)
Subjects with TEAEs leading to study discontinuation	7 (10.9)	19 (27.9)
Subjects with at least 1 drug-related TEAE	8 (12.5)	7 (10.3)
Subjects with at least 1 SAE	37 (57.8)	38 (55.9)
Subjects with at least 1 drug-related SAE	1 (1.6)	1 (1.6)
TEAEs affecting at least 10% of safety population		
Hypokalaemia	11 (17.2)	7 (10.3)
Diarrhoea	10 (15.6)	9 (13.2)
Vomiting	8 (12.5)	2 (2.9)
Anaemia	7 (10.9)	5 (7.4)
Septic shock	6 (9.4)	8 (11.8)
Acute kidney injury	4 (6.3)	8 (11.8)
Urinary tract infection	1 (1.6)	7 (10.3)

The safety population included all subjects who had received ≥1 dose of study drug.

**Figure 1.**
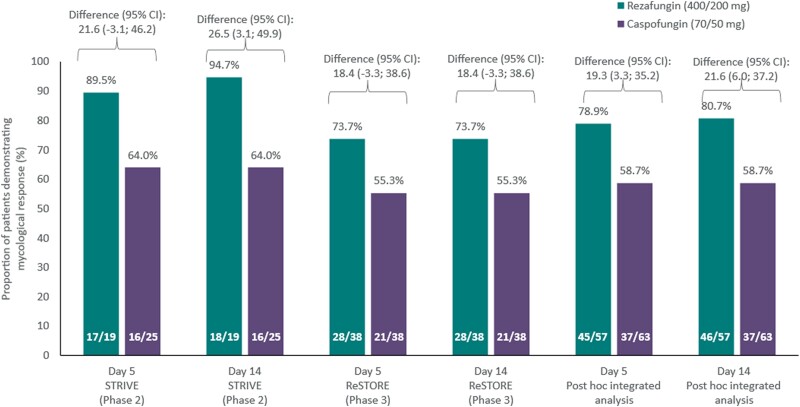
Analysis regarding mycological response at Days 5 and 14 in candidaemia/invasive candidiasis patients aged ≥65 years included in the STRIVE (Phase 2) and ReSTORE(Phase 3)clinical trials and integrated analysis of STRIVE/ReSTORE data (mITT population). All analyses were conducted using the mITT population, which included all subjects with a mycological diagnosis of candidaemia and/or invasive candidiasis within 96 h of randomization who received ≥1 dose of study drug.

## References

[1] Dekkers BGJ, Veringa A, Marriott DJE, et al Invasive candidiasis in the elderly: considerations for drug therapy. Drugs Aging 2018; 35: 781–9. 30047069 10.1007/s40266-018-0576-9 PMC6105183

[2] Thompson GR, Soriano A, Skoutelis A, et al Rezafungin versus caspofungin in a Phase 2, randomized, double-blind study for the treatment of candidemia and invasive candidiasis: the STRIVE trial. Clin Infect Dis 2021; 73: e3647-55. 32955088 10.1093/cid/ciaa1380 PMC8662762

[3] Thompson GR 3rd, Soriano A, Cornely OA et al Rezafungin versus caspofungin for treatment of candidaemia and invasive candidiasis (ReSTORE): a multicentre, double-blind, double-dummy, randomised phase 3 trial. Lancet 2023; 401: 49–59. 36442484 10.1016/S0140-6736(22)02324-8

